# Elevation of free triiodothyronine (fT3) levels by *Plasmodium falciparum* independent of thyroid stimulating hormone (TSH) in children with uncomplicated malaria

**DOI:** 10.1002/jcla.25013

**Published:** 2024-01-25

**Authors:** Henrietta Kwansa‐Bentum, Enoch Aninagyei, David Adedia, Nii Korley Kortei, Adjoa Boakye Agyemang, Clement Okraku Tettey

**Affiliations:** ^1^ Department of Biomedical Sciences, School of basic and Biomedical Sciences University of Health and Allied Sciences Ho Ghana; ^2^ Department of Basic Sciences, School of basic and Biomedical Sciences University of Health and Allied Sciences Ho Ghana; ^3^ Department of Nutrition and Dietetics, School of Allied Health Sciences University of Health and Allied Sciences Ho Ghana

**Keywords:** free tetraiodothyronine, free triiodothyronine, isolated fT3 thyrotoxicosis, *Plasmodium falciparum*, thyroid stimulating hormone, uncomplicated malaria

## Abstract

**Background:**

Malaria parasites have a devastating effect on the infected host. However, there is a paucity of data on the effect of *Plasmodium falciparum* on thyroid hormones.

**Methods:**

This case–control study (1:1) involved children <16 years of age with uncomplicated malaria. Hematological parameters were determined using the URIT‐5380 hematology analyzer (China). Later, levels of thyroid hormones, namely free triiodothyronine (fT3), free tetraiodothyronine (fT4), and thyroid‐stimulating hormone (TSH), were determined using human ELISA kits (DiaSino ELISA kit, Zhengzhou, China).

**Results:**

Ninety children with malaria and ninety matched control group were studied. Overall, compared to the control group, lower TSH (3.43 ± 1.25 vs. 3.84 ± 1.34, *p* = 0.035) and elevated levels of fT3 levels (5.85 ± 1.79 vs. 3.89 ± 1.19, *p* < 0.001) were observed in patients with malaria. However, fT4 levels were comparable between cases and control group (16.37 ± 2.81 vs 17.06 ± 3.5, *p* = 0.150). Free T3 levels were significantly higher in children <10 years (*p* < 0.001) and higher among male children with malaria (*p* < 0.001). Overall, there was a significant positive relationship between parasite counts and fT3 (*R* = 0.95, *p* < 0.001). Furthermore, body temperature was positively correlated with fT3 (*R* = 0.97, *p* < 0.001).

**Conclusions:**

Isolated fT3 thyrotoxicosis was observed in falciparum malaria, especially in children <10 years and male malaria patients, independent of TSH. This observation could explain the severity of malaria in children.

## Graphical abstract

Malaria parasites have devastating effect on the infected host. In spite of these, there is a paucity of data on the effect of *Plasmodium falciparum* on thyroid hormones. This study reported isolated fT3 thyrotoxicosis in falciparum malaria, especially in children ≤10 years and male patients with malaria, independent of TSH.

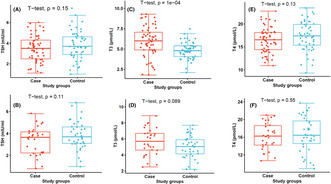

## 1 INTRODUCTION

In Ghana and generally in sub‐Saharan Africa, malaria continues to cause a significant number of morbidity^1^ and mortality^2^ especially in children.^3^ Children, particularly, under 15 years of age are disproportionately affected by malaria,^4^ although when segregated by age, children below 5 years of age suffer the most from malaria in Ghana^5^, ^6^, ^7^ and elsewhere.^8^, ^9^, ^10^ There are varied prevalence rates of malaria in Ghana, among all age groups. In the Ahafo region of Ghana, a prevalence rate of 22.8% was reported.^11^ The prevalence rates in the Greater Accra, Eastern, Central, and Ashanti regions are 15.1%,^12^ 66%,^13^ 38.2%,^1^ and 32%,^14^ respectively. The transmission of malaria in Ghana has been linked to having low or no formal education, a low family wealth index, a rural resident living near stagnant water, no history of residual indoor spraying and infrequent use of a long‐lasting insecticide‐treated net (LLITN) during the last night, as well as living in a house surrounded by cultivated land.^15^

Malaria parasites have been reported to have a devastating effect on the infected host, both at the organ and molecular levels. On blood molecules, *P. falciparum* has been found to directly hemolyze red blood cells.^16^ In children, malaria parasites are known to have a deleterious effect on the gastro‐intestinal system^17^ and the brain.^18^ Furthermore, the bone marrow,^19^ kidneys,^20^, ^21^ and the liver^22^ are organs affected by the malaria parasites.

Despite the pathological effect of malaria parasites on body organs, there is a paucity of data and information on the effect of parasites on thyroid hormones. Thyroid hormones (triiodothyronine and tetraiodothyronine) are synthesized in the thyroid gland by iodination and coupling of two molecules of the amino acid tyrosine. This process is dependent on an adequate supply of iodide.^23^ The thyroid gland actively absorbs iodide under the control of thyroid stimulating hormone (TSH), which is produced by the anterior pituitary gland.^24^ Synthesis of thyroid hormones is initiated by rapid conversion of iodide into iodine within the thyroid gland, which is catalyzed by thyroid peroxidase (TPO).^25^ The iodination of tyrosine residues takes place to form mono‐iodotyrosine (MIT) and diiodotyrosine (DIT) mediated by the enzyme TPO. Iodotyrosines are coupled to form T4 (DIT and DIT) and T3 (DIT and MIT), which are stored in the thyroid cells.^26^ The free forms of these hormones, fT3 and fT4, are biologically active compared with the bound forms.^27^ To date, it is not clear what role thyroid hormones play in the development of fever in patients with malaria. In 1990, a study reported thyroid hormone levels among patients with malaria.^28^ However, current data are lacking in the subject area. Therefore, this study was conducted to compare the levels of TSH, fT3, and fT4 in children with malaria and those without malaria. Additionally, the relationship between thyroid hormones and parasitemia, body temperature, and hematological parameters was determined.

## 2 METHODS

### 2.1 Study design, participant selection, and inclusion criteria

This case–control study was carried out in patients suspected of malaria recruited from the Ga North Municipal Hospital, Ofankor, Greater Accra region of Ghana (Latitude: 5.706980; Longitude: −0.300200). The hospital is the only referral public health facility in the municipality. The hospital receives patients referred from about 30 smaller public and private health facilities. The patients recruited for this study were selected from March to August 2019. The malaria patients included in this study were children under 16 years of age, having falciparum malaria with parental consent. To be included in the control group, patients under 16 years of age and negative for both microscopy and the malaria rapid diagnostic test were selected, with parental consent. Additionally, participants included in this study were tested for HIV and viral hepatitis; only those with negative results were qualified to be studied. Study participants were purposively selected. This was done by selecting participants in each arm of the study until the minimum sample size was exceeded.

### 2.2 Sample size determination

The sample size was calculated based on the formula to determine the sample size for case–control studies published by Jaykaran Charan.^29^

$$\text{Sample size}=\frac{r+1}{r}\frac{\left(p^{*}\right)\left(1-p^{*}\right){\left(Z_{\beta }+Z_{\alpha /2}\right)}^{2}}{{\left(p_{1}-p_{2}\right)}^{2}}$$

where: *r* = ratio of control to cases (1:1); p* = mean proportion of children under 16 years of age with malaria = (proportion in cases + proportion in control)/2; *Z*_β_ = Standard normal variate for power of 80% (0.84), *Z*_a/2_ = Standard normal variate for level of significance (1.96); *p*1 – *p*2 = Effect size where p1 is proportion of anemia in malaria in cases and p2 is proportion of malaria in control group. The proportion of malaria cases contributed by patients under 16 years, as previously reported in the Greater Accra region of Ghana was 89.3%.^30^ Due to the unavailability of data on the effect of malaria parasites on thyroid functions, the size of the effect was determined on the basis of hematological parameters. In the Volta region of Ghana, a contiguous region to the region in which this study was conducted, 53.8% of children with malaria were found to have malaria^31^ while proportion of children under 16 years of age without anemia in the same region was 30.8%.^32^ Based on these parameters, the minimum sample size in each arm of the study was calculated to be 27.

### 2.3 Clinical presentation of patients

The clinical presentation of each patient was collected from the parent or guardian accompanying the child to seek medical care. This was done on direct questioning. The clinical presentations evaluated were fever, nausea, vomiting, diarrhea, and pallor. The assessment was performed by a pediatrician or the attending physician.

### 2.4 Sample collection, processing, and storage

Whole blood samples were collected by the resident phlebotomist at the hospital. Approximately 4 mL of blood sample were collected from each child, dispensed in an EDTA tube for immediate hematological analysis. Hematological parameters were determined using the URIT‐5380 5‐Part‐Diff Auto Hematology Analyzer (China). Plasma was separated from the remaining blood samples and stored at −30°C prior to enzyme immunoassays.

### 2.5 Laboratory analysis for malaria parasites

Patients suspected of malaria were initially tested for malaria using the CareStart malaria rapid diagnostic test (mRDT) kit (Access Bio, USA). This mRDT kit detected the *Plasmodium* parasite antigen, *Plasmodium falciparum* histidine‐rich protein 2 (Pfhrp2). The test was done as previously described.^5^ All samples initially tested positive for Pfhrp2, were subjected to microscopy and parasitemia quantified. This methodology has previously been described.^5^

### 2.6 Determination of thyroid hormones

The levels of thyroid hormones, namely, TSH, fT3, and fT4, were determined from the room‐temperature plasma samples. The hormones were measured using the DiaSino ELISA kit (Zhengzhou, China). The measurements were done as directed by the manufacturer. TSH, fT3, and fT4 measurements followed a similar protocol. The antibody‐coated wells (TSH, fT3, and fT4) were brought to room temp (25°C). Exactly, 50 μL of TSH, fT3, and fT4 standards were dispensed into the first seven wells of the plates. Followed by plasma samples (50 μL) in the remaining required wells. Subsequently, 100 μL of ready‐to‐use enzyme conjugate reagent were added to all wells (both standards and samples). The wells were shaken for approximately 10 to 30 s, covered with plate sealer, and incubated for 60 min at room temperature (25°C). After incubation, the wells were washed three times with about 300 μL of 1× wash buffer. The well contents were discarded after which 100 μL of TMB substrate was added to all wells. And incubate for 15 min at room temperature, after which 50 μL of stop solution was added to all wells, gently shaken to mix the solution. The absorbance of the final yellow color was read at 450 nm (with plate correction at 630 nm) within 15 min after the stop solution was added. The manufacturer standard concentration values of THS, fT3, and fT4 and their respective absorbance were used to create a standard curve from which the sample concentrations were derived.

### 2.7 Data and statistical analysis

The R statistical package was used for the statistical analysis. The difference between unpaired continuous data was determined by the *t*‐test when the samples were normally distributed; else a Mann–Whitney test was applied, which was presented in table form and, in some cases, as boxplots. The correlations between variables were also determined with scatter plots and correlation coefficients. In all analyzes, *p*‐values < 0.05 were considered statistically significant.

## 3 RESULTS

A total of 180 patients, comprising 90 children with uncomplicated malaria (cases) and 90 matched control group, were studied. The number of cases less than or equal to 10 years was 60, while that of the control group was 59, whereas the rest were over 10 years in both participants category. However, in both cases and control group, females dominated, the differences were not significant (*x* ^2^ = 0.561, *p* = 0.454). The mean ages of the cases and control group were 8.8 and 9.6 years (*p* = 0.07), respectively. The median age for the cases was 8.5 while that of the control group was 9 years. The other parameters are shown in Table 1.

**TABLE 1:** Comparing age and gender among cases and control group.

Parameter	Case *N* (%)	Control *N* (%)	Chi‐squared (*p*‐value)
Age			
≤10 years	60 (53.6)	59 (65.6)	0.025 (0.875)
>10 years	30 (44.1)	31 (34.4)	
Central tendency (years)			
Mean	8.8	9.6	*t* = 1.8 (*p* = 0.07)
Standard deviation	2.9	2.7	
Median	8.5	9	
Quartiles			
1st	6	8	
2nd	8.5	9	
3rd	11	11	
Gender			
Male	43 (47.8)	38 (42.2)	0.561 (0.454)
Female	47 (52.2)	52 (57.8)	

Compared with control subjects, patients with clinical malaria reported higher temperatures (*p* < 0.001), leukocyte count (*p* < 0.001), eosinophils (*p* < 0.001), basophils (*p* < 0.001), mean platelet volume (*p* < 0.001), and platelet large cell ratio (*p* < 0.001). Clinical malaria patients reported lower erythrocytes (*p* < 0.001), hemoglobin (*p* < 0.001), hematocrit (*p* < 0.001), PLT (*p* < 0.001), and plateletcrit (*p* < 0.001) compared with control subjects (Table 2). When segregated by sex among malaria patients, leukocyte count was significantly lower for males (7.01% ± 1.65) than in females (8.24 ± 1.88) (*p* = 0.002). Among the control group, males reported significantly higher erythrocytes (5.46 × 10^12^/L ± 0.57, *p* = 0.010), hemoglobin (13.61 g/dL ± 1.76, *p* = 0.008) and hematocrit (45.03% ± 4.86, *p* = 0.002) than the female mean erythrocyte (5.10 × 10^12^/L ± 0.71), hemoglobin (12.02 g/dL ± 2.38), and hematocrit (41.18% ± 6.13), respectively.

**TABLE 2:** Differences in the levels of hematological parameters and temperature between cases and control group.

Variables	Case (*N* = 90)	Control (*N* = 90)	*p*‐Value
	Mean ± SD	Mean ± SD	
Temperature (°C)	38.78 ± 1.39	36.17 ± 1.05	<0.001
Hematological parameters			
TWBC (×10^9^/L)	7.65 ± 1.87	5.42 ± 1.77	<0.001
Neutrophils (%)	64.67 ± 7.74	66.71 ± 5.69	0.219
Lymphocytes (%)	27.8 ± 8.25	39.54 ± 32.15	0.206
Eosinophils (%)	3.45 ± 1.09	2.02 ± 1.64	<0.001
Monocytes (%)	4.53 ± 1.72	14.06 ± 23.36	0.101
Basophils (%)	0.4 ± 0.16	0.24 ± 0.15	<0.001
Erythrocytes (×10^12^/L)	3.66 ± 0.53	5.25 ± 0.68	<0.001
Hemoglobin (g/dL)	9.73 ± 1.7	12.69 ± 2.27	<0.001
Hematocrit (%)	29.87 ± 4.34	42.81 ± 5.92	<0.001
Mean cell volume (fL)	81.5 ± 10.63	81.08 ± 6.3	0.747
Platelet (9 10^9^/L)	151.35 ± 66.52	236.57 ± 72.27	<0.001
Mean platelet volume (fL)	8.54 ± 1.1	9.65 ± 1.06	<0.001
Plateletcrit (%)	0.16 ± 0.1	0.2 ± 0.07	<0.001
Platelet large cell ratio (%)	22.84 ± 4.18	17.11 ± 5.1	<0.001

Analysis of thyroid hormones revealed that TSH levels were significantly lower in malaria cases compared with control group (3.43 ± 1.25 vs. 3.84 ± 1.34, *p* = 0.035) while elevated levels of fT3 were observed in malaria cases (5.85 ± 1.79 vs. 3.89 ± 1.19, *p* < 0.001). Despite these variations, fT4 levels in the cases did not differ significantly from the levels observed in the control group (16.37 ± 2.81 vs. 17.06 ± 3.5, *p* = 0.150), although a numerically higher mean level was observed in the control group (Table 3).

**TABLE 3:** Thyroid hormone levels in cases and control group.

Thyroid hormones	Case (*N* = 90)	Control (*N* = 90)	*p*‐Value
	Mean ± SD	Mean ± SD	
TSH (mIU/mL)	3.43 ± 1.25	3.84 ± 1.34	0.035
fT3 (pmol/L)	5.85 ± 1.79	3.89 ± 1.19	<0.001
fT4 (pmol/L)	16.37 ± 2.81	17.06 ± 3.5	0.150

**FIGURE 1:**
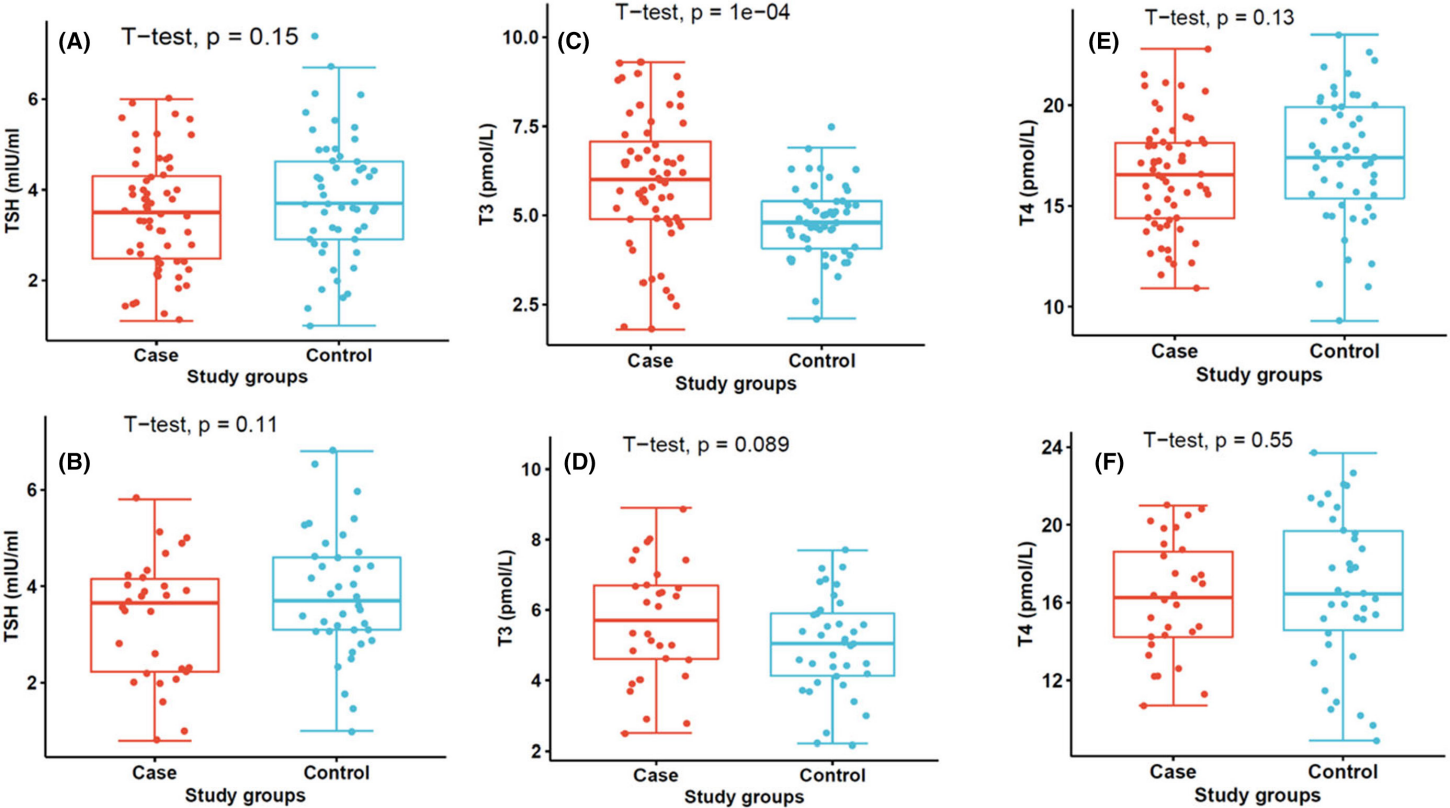
Stratification of thyroid hormones by age. (A) TSH levels in malaria cases <10 years, (B) TSH levels in malaria cases >10 years, (C) fT3 levels in malaria cases>10 years, (D) fT3 levels in malaria cases >10 years, (E) fT4 levels in malaria cases <10 years, (F) fT4 levels in malaria cases >10 years.

**FIGURE 2:**
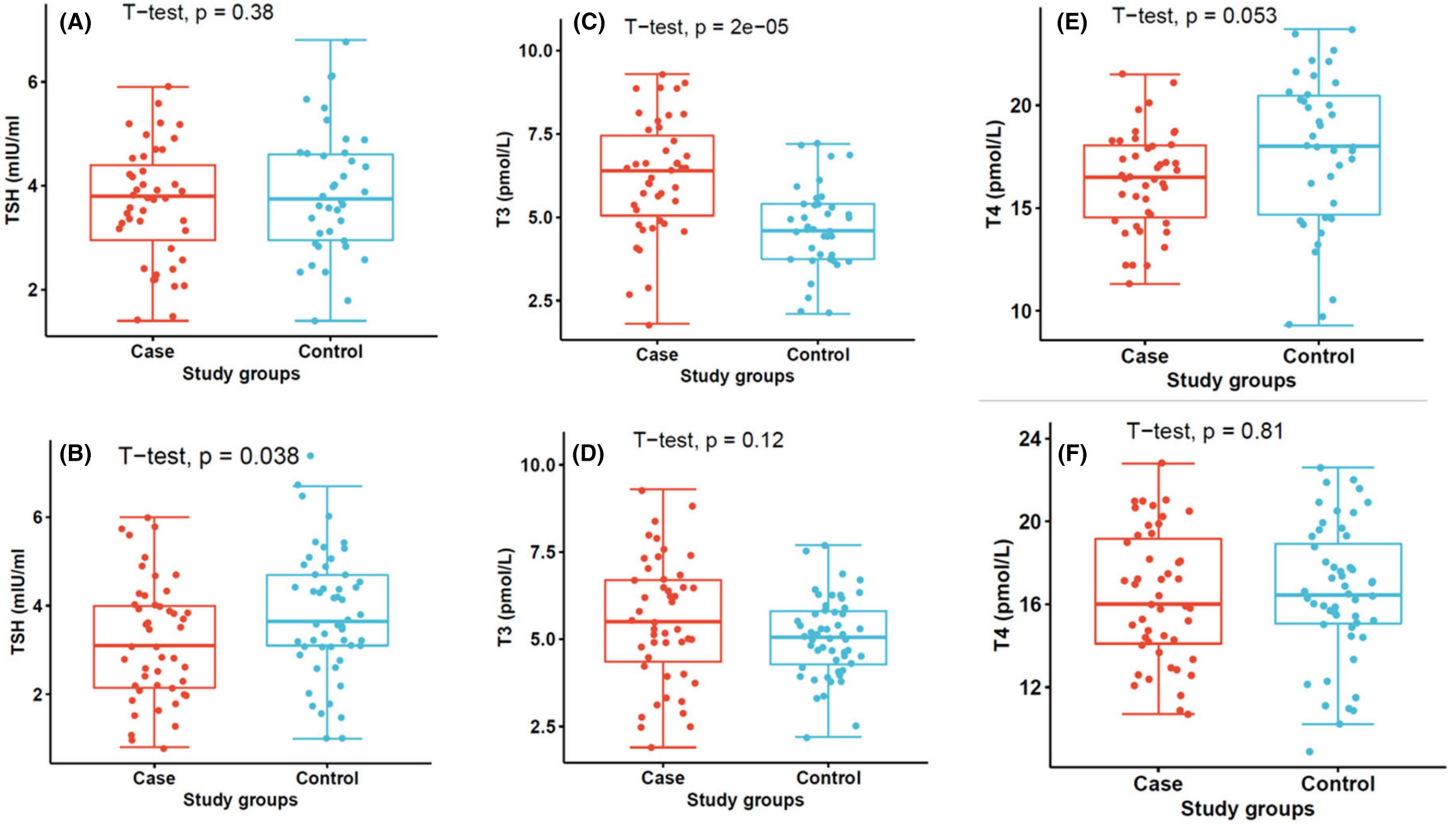
Stratification of thyroid hormones by sex. (A) TSH levels in male malaria cases, (B) TSH levels in female malaria cases, (C) fT3 levels in male malaria cases, (D) fT3 levels in female malaria cases, (E) fT4 levels in male malaria cases, (F) fT4 levels in female malaria cases.

Although the levels in mean TSH levels differed significantly between the two study groups, when segregated by age, the levels were comparable between the two age groups (10 years [*p* = 0.15] and <10 years [*p* = 0.11]) (Figure 1) among patients with malaria, but the levels observed in females with malaria were lower than their corresponding control group (*p* = 0.038) (Figure 2). Furthermore, in general, fT3 levels were higher in malaria cases than in the control group, but when stratified by age, the levels observed in malaria patients <10 years were significantly higher (*p* < 0.01) than in patients over 10 years (*p* = 0.089) (Figure 1). However, gender did not have an effect on fT3 levels (Figure 2). Finally, fT4 levels were comparable in age and gender disparity (Figures 1 and 2).

Table 4 presents the association of clinical presentations of malaria patients with extremes of fT3 or TSH. The clinical presentations of the patients were observed to be comparable regardless of fT3 or TSH levels. Despite this comparability, the frequencies of the reported clinical presentations were higher in children with significantly higher fT3 [chills (86.2% vs. 84%), fever (87.7 vs. 84%), vomiting (76.9% vs. 40%), diarrhea (16.9% vs. 12%), nausea (80% vs. 44%), and pallor (46.2% vs. 36%)] compared to children without elevations of fT3. However, a lower level of TSH resulted in marginally higher frequencies of chills (85.15 vs. 79.1%) and nausea (70.3% vs. 69.8%) but not fever, vomiting, diarrhea, and pallor.

**TABLE 4:** Association of TSH and fT3 levels with malaria clinical presentation.

	Significant difference in fT3 level			Significant difference in TSH level		
	Age groups			Gender		
Clinical presentations	≤10 year^a^ *N* = 65	>10 years^b^ *N* = 25	*p*‐Value	Males^c^ *N* = 43	Females^d^ *N* = 47	*p*‐Value
Chills	56 (86.2%)	21 (84%)	0.942	34 (79.1%)	40 (85.1%)	0.815
Fever	57 (87.7%)	21 (84%)	0.901	38 (88.4%)	40 (85.1%)	0.903
Vomiting	50 (76.9%)	10 (40%)	0.115	29 (67.4%)	31 (66.0%)	0.948
Diarrhea	11 (16.9%)	3 (12%)	0.618	8 (18.6%)	6 (12.8%)	0.514
Nausea	52 (80%)	11 (44%)	0.139	30 (69.8%)	33 (70.2%)	0.984
Pallor	30 (46.2%)	9 (36%)	0.578	19 (44.2%)	20 (42.6%)	0.921

^a^ Significantly higher fT3 level.

^b^ Insignificantly higher fT3 level.

^c^ Significantly lower TSH level.

^d^ Insignificantly lower TSH level.

**FIGURE 3:**
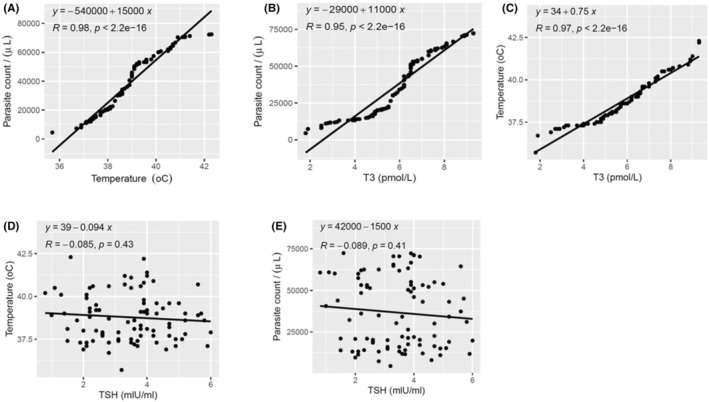
Relationships between temperature and parasite count and significant thyroid hormones. (A) Correlation between parasite counts and temperature; (B) correlation between parasite counts and fT3; (C) correlation between temperature and fT3; (D) correlation between temperature and TSH; (E) correlation between parasite count and TSH.

There was a significant positive relationship between parasite counts and temperature (*R* = 0.98, *p* < 0.001) and fT3 (*R* = 0.95, *p* < 0.001). Additionally, the temperature recordings of the patients were positively correlated with fT3 (*R* = 0.97, *p* < 0.001). There were weak negative correlations between TSH and temperature (*R* = −0.085, *p* = 0.43) and parasite count (*R* = −0.089, *p* = 0.041) (Figure 3).

## 4 DISCUSSION

This study explored the pathological effect of *P. falciparum* parasites on thyroid physiology and functions, using thyroid function hormones, namely triiodothyronine (T3), tetraiodothyronine (T4), and thyroid‐stimulating hormones (TSH), as indicators. Free forms of these hormones were measured due to their potent biological activity compared to bound or the total forms.^33^ Although both biomolecules can trigger biological effects, T3 is considered the biologically active thyroid hormone that binds to thyroid hormone receptors, while T4 is a prohormone that must be converted to T3 to initiate signaling and gain biological activity.^34^

To explore these relationships, children with malaria and those without malaria were comparable in terms of age and gender distributions. This study confirmed the direct relationships between malaria parasitemia and fever^21^, ^35^, ^36^ and derangements of hematological parameters^30^, ^37^, ^38^, ^39^ as has been widely published. The mean temperature reading of the malaria patients was 38.78 ± 1.39°C with a significant reduction in erythrocytes, hemoglobin, hematocrit, platelet levels, mean platelet volume, and plateletcrit. Significant elevation in platelet large cell ratio indicated effective platelet activity^40^ despite low level of platelets count. Surprisingly, mean cell volume was found to be not significantly reduced among patients with malaria.

A Caucasian study done by Ferraro et al.^41^ published the normal human ranges of thyroid hormones. Compared with the mean values observed in this study, the levels of TSH, fT3, and fT4 for both cases and the control group were within normal human ranges. However, TSH levels were significantly lower in children with malaria compared to those without malaria, while fT3 levels were also significantly higher in cases compared with the control group. However, fT4 levels between cases and control group did not differ significantly. Significantly lower levels of TSH were observed in the cases corresponding to significantly higher levels of T3 due to the known negative relationship that exist between these hormones.^42^ The same relationship exists between TSH and T4,^42^ therefore, higher level of fT4 was expected to be seen in malaria cases, but the reverse was observed.

Despite this observation, the significant elevation of fT3 in malaria cases cannot be overlooked, as fT3 has been reported to have higher biological activity than fT4.^42^, ^43^ The biomechanism that underpins fT3 elevation in malaria cases has not yet been established. According to the findings of this study, thyroid hormone levels in relation to *P. falciparum* infection were consistent with isolated fT3 thyrotoxicosis. Isolated fT3 thyrotoxicosis is observed in conditions where fT4 and TSH levels are in the normal human range with elevated fT3 level.^44^ In contrast to publication by,^41^ the mean level of fT3 observed in the malaria cases was above the normal Ghanaian level of 2.73–4.13 pmol/L.^45^ In isolated fT3 thyrotoxicosis, fatigue, lethargy, and weight loss have been reported,^46^ clinical presentations that are commonly observed in about 74% of children with malaria.^5^ However, it is not clear whether these symptoms observed in malaria cases could be attributed to elevated fT3. Despite the overall significantly elevated fT3 observed in malaria cases, mean levels were significantly higher (6.0 pmol/L) in children up to 10 years of age compared to those over 10 years of age (5.1 pmol/L). However, this elevation did not have an impact on the clinical presentations observed in these cohorts, although more children in the 10‐year age group than in the >10‐year group presented chills, fever, vomiting, diarrhea, nausea, and pallor. It was also observed that TSH levels were significantly lower if females than males, despite the overall significantly lower levels observed in malaria patients compared with control group. However, gender differences in TSH levels were not associated with the clinical presentation of the patients.

Despite the lack of association between fT3 and fever, there was a strong and positive correlation between temperature readings and fT3. Additionally, a strong positive correlation between parasite count and fT3 was observed in this study. This correlation is too strong to ignore and can therefore mean that malaria parasitemia may play a significant role in elevated fT3. In addition, the malaria parasite count was strongly correlated with the temperature reading. An association which has been previously established.^47^ It is known that fever in malaria occurs as a result of rupture of parasite‐infected red cells with new red cells being infected; however, fT3 contributes to thermogenesis, a mechanism that needs to be properly investigated and established. Temperature recordings in malaria cases and parasite count were negatively correlated with TSH levels, without significant association. This lack of association does not suggest the direct role of malaria parasites in inhibiting TSH release from the anterior pituitary gland. Therefore, the effect of malaria parasites on fT3 is independent of TSH.

A previous study has published thyroid hormone levels in patients with severe falciparum malaria among the Thais^28^ and reported depressed thyroid function regarding total T4 compared with control group. T3 was not measured in that study. However, this study observed that fT4 was lower compared with the control group, although insignificant. It is possible that if the study by Davis et al. (1990) had measured free or T3, elevated levels would have been observed. This is because the malaria parasite was found to increase the iodine concentration in the thyroid, in an earlier study,^48^ which ultimately leads to increase in the formation of thyroid hormones.

### 4.1 Conclusions

TSH and fT3 levels were altered in children with falciparum malaria. However, the elevation of fT3 was significant in children up to 10 years of age, while women were significantly associated with lower TSH levels. These hormonal changes could be the reason why children under 10 years of age and women are severely affected by malaria. It has previously been published that mortality rates due to malaria in women are significantly higher compared with men. In addition, women with malaria have a lower hemoglobin level and a higher incidence of convulsions than men with malaria. Furthermore, shock in women with malaria has been found to be significantly higher compared to male patients with malaria.^49^ For these reasons, prompt treatment for malaria in children under 10 years of age, especially female patients, can be lifesaving.

### 4.2 Limitations of the study

Despite the strict inclusion criteria used for this study, preexisting non‐communicable diseases, nutritional status, coexisting infections, and previous medications could affect the results reported in this study.

## FUNDING INFORMATION

This study was funded from the authors' own resources. No external funding was received.

## CONFLICT OF INTEREST STATEMENT

The authors declare that there is no conflict of interest to declare.

## Data Availability

All data obtained in this study have been presented in the manuscript.
